# Feasibility demonstration of a massively parallelizable optical near-field sensor for sub-wavelength defect detection and imaging

**DOI:** 10.1038/srep26172

**Published:** 2016-05-17

**Authors:** Mahkamehossadat Mostafavi, Rodolfo E. Diaz

**Affiliations:** 1 School of Electrical, Computer, and Energy Engineering, Arizona State University, Tempe, AZ, 85281, USA

## Abstract

To detect and resolve sub-wavelength features at optical frequencies, beyond the diffraction limit, requires sensors that interact with the electromagnetic near-field of those features. Most instruments operating in this modality scan a single detector element across the surface under inspection because the scattered signals from a multiplicity of such elements would end up interfering with each other. However, an alternative massively parallelized configuration, capable of interrogating multiple adjacent areas of the surface at the same time, was proposed in 2002. Full physics simulations of the photonic antenna detector element that enables this instrument, show that using conventional red laser light (in the 600 nm range) the detector magnifies the signal from an 8 nm particle by up to 1.5 orders of magnitude. The antenna is a shaped slot element in a 60 nm silver film. The ability of this detector element to resolve λ/78 objects is confirmed experimentally at radio frequencies by fabricating an artificial material structure that mimics the optical permittivity of silver scaled to 2 GHz, and “cutting” into it the slot antenna. The experimental set-up is also used to demonstrate the imaging of a patterned surface in which the critical dimensions of the pattern are λ/22 in size.

The need to resolve sub-wavelength features at optical frequencies arises in many areas of Physics and Engineering, and is particularly critical for the semiconductor industry. Thus, the international technology roadmap for semiconductors (ITRS) has listed among its metrology *difficult challenges* the detection and sizing of very small particles, with particle detection limits of 11 nm required now, and down to 8 nm in 2017. Particularly challenging is the silicon dioxide substrate used for silicon on insulator (SOI) technology because its enhanced optical reflection and surface quality impact the sensitivity of traditional optical methods while surface charging effects interfere with electron beam methods^1^.

At these sizes, the highest throughput defect detection technology available today, visible laser scatterometry, is not viable because such particles are well below the diffraction limit (at most λ/40 in size). Therefore, deep ultraviolet (DUV) scatterometry (in a vacuum) or scanning microscopy approaches such as scanning electron microscopy (SEM) or near-field optical scanning microscopy (NOSM) have been considered the only alternatives. But, for the latter to work with high throughput, the near-field sensors must be implemented into massively parallel arrays^2^. The instrument must then explicitly deal with the problem of coupling of signals between sensing tips and the shadowing of signals by adjacent tips.

However, a hybrid alternative was proposed in 2002^3^,^4^,^5^ that would enable the use of cost effective and highly robust visible lasers to interrogate a massively parallel array of near-field antenna sensors that, by construction, cannot obscure each other. This approach called the Wave Interrogated Near-Field Array (WINFA) is illustrated in Fig. 1. It consists of an array of photonic (plasmonic) antennas that is scanned above the surface to be inspected while being illuminated by a laser beam. An imaging detector collecting the scattered laser signal from the array can identify the antenna element of the array that has crossed over a defect on the surface and, with subsequent processing, identify the character of the defect (metal or dielectric) and whether it is on the surface or under it^4^,^5^.

## The Wave Interrogated Near-Field Array principle

The WINFA exploits two physical phenomena to attain its large-area sub-wavelength detection capabilities. First, by using holographic filtering in the far field, the change in the scattering signature of one antenna of the array automatically identifies that antenna in the image of the array. The way this works is as follows: Consider an array of point (dipole) scatterers, suspended over a perfect, defect free, substrate and illuminated with the interrogating laser. It is well-known that the far-field scattering pattern of that array is the Fourier transform of its aperture (the aperture being the arrayed collection of its point scatterers). Using standard optical holography techniques, that scattering pattern can be recorded as a hologram and then inverted. The result is an all-stop filter that when superimposed on the scattering signal from the perfect array lets no light through. However, if the scattered signal from one of the array elements is changed in amplitude or phase the difference passes through the hologram. Then, an optical inverse Fourier transform of the signal that permeates through the filter recreates an image of the plane of the array, where the only “lit” element is the element whose signature changed. A similar technique is the basis of optical interferometry non-destructive evaluation of structures under deformation^6^, where it is capable of detecting displacement contours separated by distances as small as λ/100.

The second phenomenon exploited by the WINFA is near-field magnification of the sub-wavelength object’s scattering signature. This magnification effect is partly due to the increased intensity of light at the near-field focus, or hot-spot, of a resonant antenna structure^7^. However, in practice, the realistic loss in noble metals limits that intensity amplification in plasmonic antennas to less than one order of magnitude; and therefore that is all that would be obtainable if the detection scheme were based on directly observing this increased scattering from the sub-wavelength object. Instead, the holographic filtering scheme described above detects *the change* in the scattering signature of the resonant plasmonic antenna, induced by the nearby object. This change can be two or more orders of magnitude greater than the object’s signature.

The simplest way to understand this phenomenon is to compare the scattering signature of a resonant electrically small object (say, a sub-wavelength noble metal sphere) with that of a non-resonant object (say, a dielectric sphere) of the same size. Following van de Hulst^8^ we know that the dominant term in the expansion of the scattering efficiency of a small sphere of radius *a,* and relative permittivity *ε, in air,* is of the form given in equation 1, where *x* is the size parameter 2π*a*/λ.
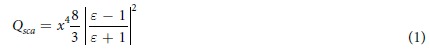


Now, if the case where the sphere is a dielectric of permittivity, ε = 2 (SiO_2_) is compared to the case where it is a noble metal (e.g. silver at 355nm) with complex permittivity ε = −2 + i0.68 (assuming equal size parameters), the ratio of the scattering cross sections of the resonant sphere (the Noble metal) to the non-resonant sphere (the dielectric) is 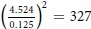.

In our case the resonant noble metal scatterer is not an electrically small sphere, but rather an antenna approximately λ/8 in length. Thus the actual ratio of scattering cross sections, when such an antenna is compared to a scatterer λ/80 in size, is in actuality 
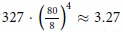
. Given this, from ref. 6 we know that holographic filtering schemes can detect phase changes equivalent to λ/100, then it is a valid assumption that the effect of the sub-wavelength particle on the antenna is of this order. That is, it changes the antenna’s signature by the factor sin^2^(2*π* · 0.01) ≈ 0.0039. This change in the antenna signature is still 3.27 · 10^6^ · 0.0039 ≈ 13,000 times larger than the signature of the particle by itself. The resulting magnification would be more than three orders of magnitude.

Obviously, the actual magnitude of the magnification factor depends on not only the size of the object, but also on its composition, its orientation relative to the electric field vector in the hot-spot, and its proximity to the antenna’s hot-spot region. Although such a magnification of the signal can be expected from the work by Watts *et al*.^4^, for precise evaluation of this magnification factor we resort to full physics simulations.

Before proceeding to the full physics evaluation, a variation to the original WINFA concept is proposed that dramatically increases its signal-to-noise ratio (SNR) and enables it to work as an imaging sensor. In the original concept the antennas in the array were assumed to be metal dipoles. However, because an array of dipoles suspended on a transparent substrate will allow a significant amount of the incident interrogating plane wave to transmit through, and impinge on the surface under test, any intentional dielectric or conducting features on that surface will scatter some of this energy into the far field and interfere with the desired signal from the dipole array. Thus, the original WINFA would excel as a defect detection instrument to inspect bare semiconductor wafers, but it could not be used for inspecting *patterned* surfaces.

Since quality assurance needs in the semiconductor industry also include the inspection of such surfaces (either lithographic masks or patterned wafers during intermediate steps in the manufacturing process^9^) and, most importantly, tools for certifying critical dimensions (CD) of the pattern itself, in this work the electromagnetic dual of the original WINFA structure is considered, where the near-field sensors are slot antennas in a noble metal layer (silver). The opacity of the silver layer to the incident illumination then shields the surface underneath, guaranteeing that the only significant interaction between the electromagnetic fields and the features on the surface occurs in the concentrated near-field at the feed-points of the antennas.

### The antenna element design

The design of the antenna element involves a series of engineering trade-offs. As the discussion relating to equation 1 illustrated, the larger the antenna, the larger can be its scattering efficiency; but at the same time the more compact the antenna is, the sharper its resonance, and the stronger is its near field. Keeping the longest dimension of the antenna below λ/2 guarantees it will operate as a dipole scatterer; but making it too small may present manufacturing difficulties if the critical details of its structure are beyond state-of-the art manufacturing (of the order of 4 nm using electron beam methods). At the same time the antenna shape, including the size of the details of its structure, gives a measure of control of the hot-spot dimensions. These trade-offs drove the design toward a folded dipole geometry similar to Watts’, resonant in the red region of the visible spectrum.

In particular, we designed a compact slot antenna, 84 nm across in a 60 nm thick silver layer, resonant at 660 nm (approximately λ/8 in size) based on Lesuffleur’s connected nano-hole construct^10^,^11^,^12^ (this “spectacles” antenna is illustrated in Fig. 2). In these structures the polarizability that dominates the resonance is not that due to the aspect ratio of the object but that due to the cusp discontinuities and sharp material transitions that occur at the junction between the holes.

Full physics simulations using the finite difference time domain (FDTD) method were used to fine tune the design. Details regarding these simulations are given in the “Methods” section. Figure 3 shows the spectrum of the scattered signal of this antenna compared to a single-hole and a connected-hole geometry.

Whereas the single-hole Fröhlich resonance (black curve) occurs at 400 nm, the two connected-holes (blue curve) exhibit two main resonances: one also around 400 nm and a stronger one at 800 nm. Adding material back inside the holes (inspired from the idea of “phasing element” in a paper by Greiser^13^) allows us tuning of the antenna to weaken the 400 nm resonance in favor of the longer wavelength resonance. Thus, in the “spectacles” antenna (red curve) the resonance at 660 nm is almost 1.5 orders of magnitude stronger than its resonance near 400 nm, and almost two orders of magnitude stronger than the resonance of a single-hole; and it lies in the desired visible red range where laser sources are plentiful. Note that in the 600 nm to 660 nm range the real part of the permittivity of silver ranges from −16 to −20, while the imaginary part is between +i2 and +i3.

The apparent wide bandwidth of the long wavelength resonances in Fig. 3 is an artifact of the computational electromagnetics method used (FDTD). It results from truncating the extremely long time history of a reverberating structure. In order to study in detail such resonant structures in the radio frequency scaled model, a frequency domain method (Ansys HFSS) will be used to avoid this artifact.

### Demonstration of the magnification effect

Using this computational model the magnification factor effect can be demonstrated. A Gaussian beam incident at approximately 50 degrees from normal was used to illuminate the slot antenna. The scattered signal exiting the top radiating boundary of the FDTD domain was integrated, Fourier transformed into the frequency domain, and stored. Then an 8 nm metallic object (not a Drüde metal since its own resonance might confound the results) was used as a candidate target of interest, placed 12 nm below the plane of the antenna and scanned along the principal axes. The resulting scattered signal was collected, Fourier transformed into the frequency domain, and to emulate the holographic filtering it was subtracted from the original stored data. As expected, the maximum signal occurs as the object crosses the hot-spot. Figure 4 shows the result.

The change (or power enhancement ∆) in the slot antenna’s signature is 20 dB greater (dB = 10log_10_(power)) than the scattering from the object by itself. That is, two orders of magnitude. The reason three orders of magnitude is not attained (as shown by Watts) is because of the intrinsic loss in silver.

Figure 4 also shows (black curve with symbols) the same result when the surface of the silver layer is given a 4 nm peak-to-peak roughness (typical of the silver interface on a smooth quartz substrate) to emphasize that this result is robust in realistic scenarios. This degree of roughness constitutes a peak-to-peak variation of the surface of the order of 1/120^th^ of a wavelength on the quartz side of the interface but of the order of 1/5^th^ of the optical penetration depth inside the silver. The minute change due to roughness in Fig. 4 might be surprising, considering that such a degree of roughness has been reported to induce up to a 25% change in the surface plasmon polariton propagation length^14^. However, the operation of this slot antenna does not depend on the propagation of surface plasmons along the surface but rather on the local oscillation of the electromagnetic fields at the surfaces of the slab, as controlled by the boundary conditions created by the slot antenna. Given that the slot antenna is 80 nm long by 40 nm wide and 60 nm deep, it appears that a + /−5% variation of its surface is not a significant perturbation to its operation.

From the discussion on the origin of the magnification effect, the size of the particle being detected and its composition must affect the degree of magnification. In general it would be expected that the smaller and the more diaphanous the particle, the stronger the magnification, since such particles have extremely small scattering cross sections to begin with (by equation 1). However, it could be argued that a larger particle may couple more effectively to the slot antenna and thus cause a larger change in its signature. The reality is that these two effects compete with each other.

To illustrate this point, the power enhancement (∆ in dB) for a variety of test particles in the FDTD simulation is shown in Fig. 5. All particles were placed at the hot-spot, such that their top surface was 12 nm from the antenna. Then their size was varied by increasing the volume. The results are plotted against the volume of the particle since the polarizability of small dielectric objects (and therefore their induced dipole moment) is proportional to their volume.

In the figure ideal metal sub-wavelength objects (not Drüde) are shown in orange and the dielectrics (with dielectric constant of 2) are in beige. The principal E-field of the antenna’s hot-spot is shown as the red arrow in the figure. This is relevant because the polarizability of a small particle also depends on the orientation of its principal axes relative to the electric field. Long particles parallel to the field scatter most strongly.

From these results it is noticeable that: (a) The dielectric objects, which are by nature weaker scatterers, gain more benefit from the antenna than the metal objects, even approaching 3 order of magnitude magnification (30 dB). (b) For objects of constant volume (A–F in Fig. 5) those objects with the longest dimension aligned with the electric field gain the greatest enhancement. (c) For objects of equal length along the electric field (G–I) those with the smaller volume gain the most enhancement. This latter effect is exacerbated by the fact that as the volume of the objects G–I increases, that additional volume moves farther and farther away from the antenna.

This concludes the review of the WINFA system and its proposed implementation using engineered slot antennas in a silver film. The next step before proceeding to the design and manufacture of the instrument is to verify the theoretical and computational work with experimental data. Since Maxwell’s equations apply across the entire electromagnetic spectrum, the experiment need not be performed at optical frequencies, but rather can be performed at any frequency. In particular to maximize the manufacturing flexibility, minimize the experimental cost, and operate with antennas and scattering objects that can be easily handled, the optical frequency range was scaled to the radiofrequency (RF) range, between 2 and 5 GHz. This way the printed circuit board (PCB) technique is used for the fabrication. The experimental set up and results obtained are now discussed.

## Results

For the scaled frequency experiment to correspond to the optical frequency reality, it is necessary to construct a material (or *effective medium*) that correctly emulates the dispersive permittivity of silver. To first order, the dispersion of a noble metal can be fitted by a single Drüde term over a finite band of frequencies, a behavior also seen at radiofrequencies in a cold plasma. As such it has been successfully mimicked by a periodic array of conducting wires, the so-called rodded effective medium^15^,^16^. However, an attempt to design such a rodded medium to mimic a 60 nm thick silver layer immediately fails in practice.

The typical effective medium restrictions are that the wire radius be small compared to the periodic unit cell (assumed cubical here, of period p), and that the periodic unit cell be small compared to the wavelength, λ. Then Brown’s formula, given as equation 2, holds:
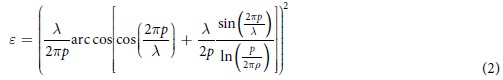
But the effective medium approximation places an additional restriction. For the electromagnetic properties of the artificial construct to be homogenized (and therefore behave as a continuum material) at least 4 unit cells are required to discretize the thickness of the slab^17^. Assuming that the scaling is from 600 nm down to 2 GHz (the largest case), the 60 nm slab will be 15 mm thick. Thus the unit cell would be 3.75 mm on the side, and equation 2 tells us that to attain a negative permittivity of −20, the wire cross sectional radius *ρ*, would have to be of the order of 25 nm. This is not practical with PCB techniques.

The inductance per unit cell required to mimic the properties of silver with a wire grid is so high that either the periodic unit has to be increased to a non-negligible fraction of the wavelength or the radius of the wire has to become vanishingly small. In fact, only if the periodic unit cell were the full thickness of the slab, 15 mm, does Brown’s equation yield a result compatible with conventional PCB manufacturing techniques: *ρ* = 1.5 mm (Of course, this value would be incorrect in practice because a single unit cell has no neighbors around it, and thus the contributions from those neighbors assumed in the effective medium model would be missing).

### The Printed Circuit Board scaled Silver medium

Therefore only two alternatives exist to create the desired scaled material: (a) increase the inductance per unit cell by building into the circuit structure inductive elements such as solenoids or chip inductors, or (b) abandon the requirement to abide by the restrictions of effective medium theory.

Option (a) in principle would allow us to choose a scaled frequency as high as 5 GHz with the material slab being 6 mm thick. However there is always a manufacturing limit imposed by the typical thinnest metal line that can be produced with conventional PCB techniques (0.07 mm). A compromise is to allow the unit cell to be larger in the transverse direction but discretize the thickness of the material (“silver”) slab into smaller sections. Then, acknowledging that the representation of the material continuum will still be coarse, the design of the structure is guided not by the effective medium permittivity but by the requirement that the artificial material slab exhibit the same transmission, T and reflection Γ, coefficients as the scaled silver slab. Once this is done, and the structure designed, it is easy to evaluate its behavior in the presence of the slot antenna using again full physics computational electromagnetics methods. Figure 6 illustrates this compromise.

The two complex parameters to be mimicked (Τ, Γ) dictate two unique admittance surfaces, Y1 and Y2 (the equations are given in the “Methods” section), as shown in Fig. 6a. The calculated reflection and transmission coefficients for the RF structure (Fig. 6b) agree with the desired scaled model result. The admittance sheets can be designed to first order by using the standard techniques of frequency selective surface design^18^. The designs then are verified using the frequency domain full physics computational tool Ansys HFSS, fine-tuned if necessary, and then, if the performance is satisfactory, PCB manufacture follows.

As expected from the Drüde nature of the material being mimicked, these admittance surfaces are inductive. Approaches to implement such inductive surfaces in PCB include the use of solenoidal and pancake geometry inductors or Cartesian grids of metal traces interconnected with chip inductor drop-in components. An example of the first approach is given in the “Methods” section.

Unfortunately, neither of these option (a) alternatives are easily manufactured. They require either “blind buried vias” to interconnect the windings of the solenoids or buried chip inductors; and though circuits with buried chip capacitors are routinely built, the size of the chip inductors needed here puts the design beyond normal PCB manufacturing techniques. Option (b) must be considered.

This raises the question: is it really necessary to emulate the volume of the negative permittivity slab to obtain an RF simulacrum of the behavior of the slot antenna in the plasmon resonant layer? The results of Luo and Maslovski^19^,^20^ suggest the answer is no. Both references show the localized plasmon resonance that occurs when one side of a negative permittivity slab is excited by a proximate electromagnetic source is a surface to surface coupling phenomenon. What really matters in that case is for the layered structure design to present the correct surface inductance to support the resonance of the slot antenna, and that the surfaces couple to each other through the antenna’s reactive near fields.

Thus the artificial construct is simplified to one dielectric slab with the same admittance sheet on both surfaces (dictated by symmetry). As was done for Fig. 6, the value of the admittance sheet is selected not based on recreating the permittivity of the desired material slab but rather its scattering coefficient. Since in this configuration only one value of admittance sheet is used, we must select which coefficient to favor, Transmission, T, or Reflection, Γ.

As the opacity of the silver slab is important to the high signal-to-noise ratio of the slot-antenna-based WINFA configuration, when faced with a choice between matching reflection or transmission properties, transmission is selected as the important parameter, as long as the surfaces are inductive and therefore capable of resonating the slot antenna. Selecting 2 GHz as the scaled frequency we find that the admittance surfaces must have an inductance between 4.3 and 5 nH per square, and be separated by 14 mm of a dielectric with relative permittivity in the 3 to 4 range.

Such values of inductance can be obtained by thin wire Cartesian grids. Again using Brown’s method^15^, the design equation is equation 3, where η is the impedance of free space (377 Ω), ε_D_ is the dielectric constant of the medium and λ_D_ is the wavelength in the medium.
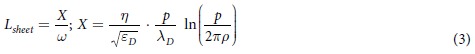


Then for a periodic unit cell of 7.6 mm on a dielectric substrate of relative permittivity 3.5, a wire radius of 0.06 mm yields 4.5 nH. The rule to convert a cylindrical wire to a planar metal strip (typical of PCB circuits) is that the perimeter must be conserved. Therefore the width of a metal strip equivalent to this wire is 0.19 mm.

These metal Cartesian grids on the top and bottom surfaces of the dielectric slab should be connected to each other by plated through vias. These prevent the propagation of trapped waves between the surfaces (since those would steal energy from the antenna resonance) and ensure that reactive near field coupling between the surfaces is what dominates the interaction in the presence of the slot antenna. (They also emulate the negative permittivity in the normal direction that the Drüde slab possesses.)

This structure was manufactured and tested. The match to the desired transmission coefficient is shown in Fig. 7.

As option (b) stated, this structure violates the rules of effective media. Yet, as the data in Fig. 7 shows it correctly mimics the desired transmission coefficient at 2 GHz and even exhibits the increased transparency that would be expected from silver as the frequency increases (except the RF structure becomes transparent at 4 GHz whereas scaled silver would reach this point at around 5 GHz). So now it remains to show that the designed “spectacles” slot antenna, when cut into this material, exhibits the desired “plasmon” resonance.

### The RF photonic antenna

With such a coarse grid, the fit of the antenna within it also strains the expectations of an effective medium. The antenna shape cuts and distorts the grid on the top and bottom faces (Fig. 8(a,b)). Yet as shown in Fig. 8c (red dashed curve), in spite of all these non-idealities, the computed transmission coefficient of a periodic array of such antennas (period = 76 mm ∗ 45.6 mm) exhibits the sharp resonance expected just below (within 10% of) 2 GHz. The field transmitted by the antennas exceeds by at least 2 orders of magnitude the field that would be transmitted through the artificial material slab.

This is, of course, the well-known *extraordinary transmission* phenomenon of apertures in Drüde metal layers, except in this case the apertures are true photonic antennas, where the resonance is not simply the result of the natural Fröhlich resonance of a sub-wavelength object but rather it has been tuned by the shape and size of the structure to produce, as seen in Fig. 8d, the desired field concentration at the open feed of the antenna. The Ansys HFSS simulations also show in perspective view the strength of the electric field normal to the surface and the principal polarization electric field extending from surface to surface (Fig. 8(e,f) respectively).

The hot-spot produced in the neighborhood of the feed is the key to this antenna’s sub-wavelength detection capabilities. On the left of Fig. 9 the computed variation of the electric field strength with distance from the antenna along the axis of the hot-spot is shown. A change of 1 decade is observed by changing the distance from 1 to 8 mm below the antenna. The cross section of the hot-spot is gauged on the right of Fig. 9 by measuring the electric field in the plane aligned with the principal polarization (the E-plane) and the plane perpendicular to that (the H-plane.) The size of the hot-spot, measured as the width at half the peak E-field value, is approximately 10 mm (λ/15) across.

### Results of the RF experiments

A 12″ by 12″ artificial medium slab following the design prescription described above was manufactured with one antenna element at its center. The test article was designed to be held by a metal frame which fits into a special-made test setup consisting of a 4′ by 4′ ground plane table under which there is an x-y computer controlled positioning stage.

To simulate the plane-wave-interrogated WINFA configuration, two broadband horn-polyrod antennas (operating from 700 MHz to 20 GHz) driven by an Agilent hp8720 vector network analyzer (VNA) are used, one as the source and one as the receiver. As in the WINFA, the receiver is positioned away for the specular reflection direction of the source. Shown in Fig. 10, the greatest sensitivity was obtained by positioning the receiver in the backscatter direction and reducing the cross talk between the antennas with a 4 inch thick absorber panel.

The poor directivity of the horn polyrod antennas at 2 GHz (the polyrods are only 3 wavelengths long at this frequency) results in a limited dynamic range in this test configuration. Software time-domain gating can be used to ameliorate the cross-talk and multipath issues, but at the same time the ability to observe the sharp resonance of the slot antenna is lost. Nevertheless, the change in the antenna signature due to perturbation by a nearby object can be observed.

Figure 11a shows the test object, a small strip of metal 5 mm wide (λ/32) at 3 mm (λ/52) below the antenna, affixed to a Styrofoam holder that is mounted on a layer of honeycomb absorber (to hide the scattering from the positioner platform below). Figure 11b shows the received signal as the object is scanned along a 120 mm long line centered on the antenna and aligned with its principal polarization. A 1 dB change on a background with about + /−0.25 dB noise is observed.

At optical frequencies there is no multipath problem and no requirement for a time domain gate because lasers are extremely high directivity illuminators with a very narrow band of operation, ideally suited to the narrow bandwidth resonance expected from the antenna. To more closely mimic this aspect of the optical environment, instead of the free space broadband sources, a waveguide source is used to directly couple to the grid and antenna. As Fig. 12 (left) illustrates, an S-band coax to waveguide transition is chosen as the source.

Though an S-Band waveguide is below cutoff at 2 GHz, the short distance between the coax probe feed inside it and the flanged aperture (7.2 cm by 3.4 cm in area) ensures that any object at the aperture plane is illuminated. The compact size of this illuminator allows us to excite the area of the grid that has the slot antenna or, if we desire, an adjacent area with no antenna. In this test set-up the measured signal is the reflection coefficient at the aperture. A very clear antenna resonance can be seen (red solid curve: a dip in the reflection coefficient because at resonance the aperture transmits the signal). By comparison, the grid alone (blue curve) strongly reflects the signal. These measurements show very little noise and confirm that our RF scaled antenna and material behave as designed: resonant just below 2 GHz, as the computational result of Fig. 8c suggested.

**Figure 1:**
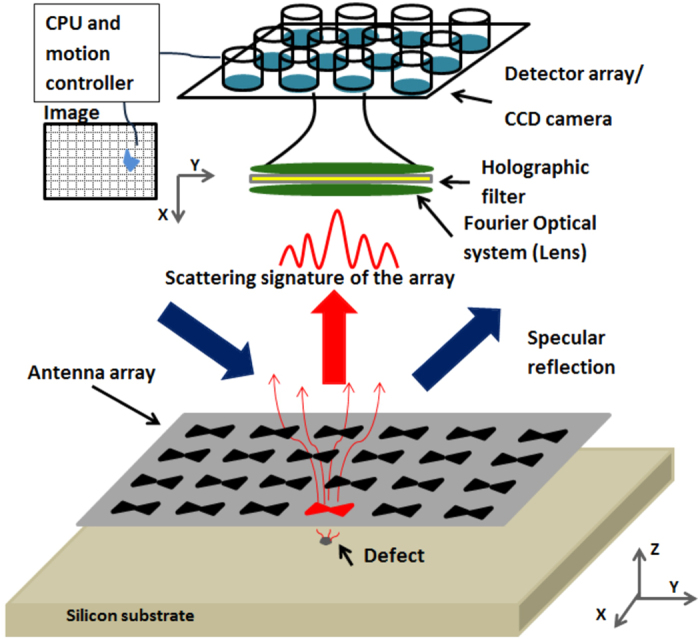
Schematic of the original WINFA system.

**Figure 2:**
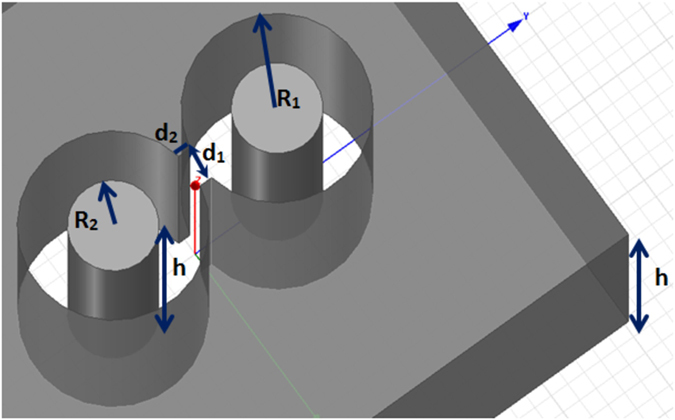
Compact slot antenna inside silver slab R_1_ = 20 nm, R_2_ = 10 nm, d_1_ = 12 nm, d_2_ = 4 nm, and h = 60 nm.

**Figure 3:**
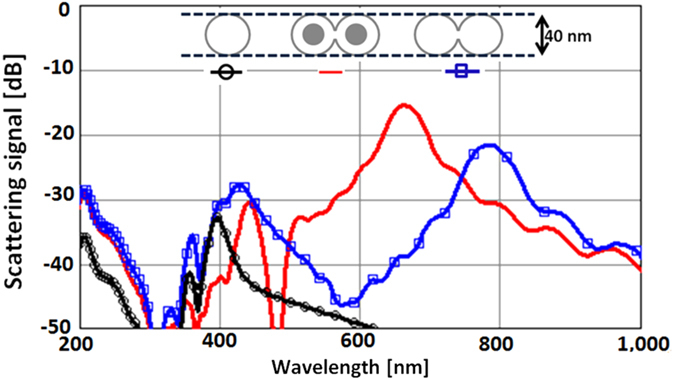
The connected-hole geometry yields compact slot antennas with very long wavelength resonance.

**Figure 4:**
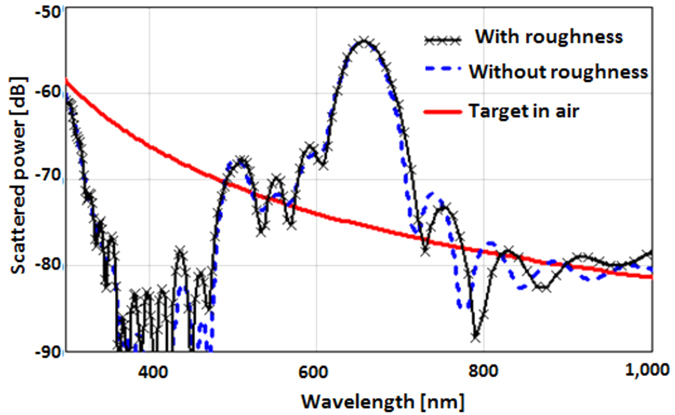
The scattered signal from an 8 nm object by itself (red) is 2 orders of magnitude lower than the change in the scattered signal of the antenna due to the object’s presence in its hot-spot, with or without roughness (black and dashed blue respectively).

**Figure 5:**
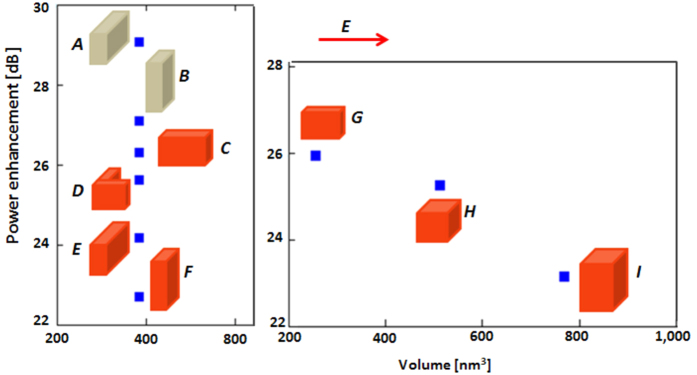
Power enhancement in dB for small particles of varying composition (red = metal) (beige = dielectric) and volume. The dimensions of the sub-wavelength objects all in *nm* are: *A*: 4*12*8; *B*: 4*8*12; *C*: 12*4*8; *D*: each section: 8*4*8; *E*: 4*12*8; *F*: 4*8*12; *G*: 8*4*8 *H*: 8*8*8; *I*: 8*8*12.

To emulate the function of the holographic filter in the WINFA optical system, we store in the network analyzer’s memory the amplitude and phase of the unperturbed antenna’s signature and implementing the complex “Math Divide” function directly record the *change signal*. The significant increase in dynamic range of this test set-up is evident in Fig. 13 (when compared with Fig. 1) where the amplitude and phase of the change signal from a λ/35 metal object scanned 3 mm below the antenna is shown. The change signal is at least 20 times stronger than the background (jitter) noise.

A very simple way to process the change signal data seen in Fig. 13, to generate both amplitude and phase images, is to integrate the absolute value of the signal just around the resonance (say, from 1.83 GHz to 1.95 GHz). The rest of the images to be shown below are processed this way.

In Fig. 14 we show the response to a dielectric object (ε_r_ ~ 2.6) 2 mm by 2 mm by 3 mm thick scanned 1.5 mm below the antenna. This λ/78 object was the smallest target attempted to be measured. The signal is now only 2 to 3 times stronger than the background noise but the antenna clearly sees it. *At the optical frequencies this would be equivalent to detecting an 8* *nm defect using red laser light.*

Finally, the properties of the hot-spot were measured. A metal square object 4 mm on the side (λ/38) was positioned at the center of the antenna and displaced from 1.5 mm to 6 mm along the z-axis. The strength of the amplitude signal is shown in Fig. 15a normalized to the maximum value (the signal at 1.5 mm below the antenna). A one decade drop over a 4 mm traverse is observed. This corresponds in the optical case to a one decade drop in the signal from a 17 nm object, moving from 6 nm to 24 nm away. This result, though consistent with that in Fig. 9 (left), cannot be compared directly because the latter is only a measure of the electric field produced under the antenna whereas the result of Fig. 16a includes the interaction between the object and the antenna.

By scanning a sharp dielectric edge under the antenna, the transverse dimensions of the hot-spot were measured as shown in Fig. 15b. A 10 mm half-field hot-spot is seen, in agreement with the computed result in Fig. 9.

Taking together all the experimental results at the RF range demonstrates the success in mimicking: (i) the properties of the noble metal slab, (ii) the resonance of the antenna, (iii) the field concentration phenomenon, and (iv) the defect detection capabilities of the proposed optical near-field sensor.

### Demonstration of the imaging capabilities

The “spectacles” antenna is similar in design to the folded dipole of Watts^4^. It combines an electric dipole mode polarized along its feed gap with a magnetic quadrupole mode (with magnetic fields flowing normal to the plane of the antenna at the apertures) due to counter-rotating electric currents along the circular rims of the spectacles. At radio frequencies, a metal object presenting a significant cross sectional area to the antenna can respond to both fields because it possesses both an electric and a magnetic polarizability. (The magnetic fields will induce eddy currents on the metal object).

Thus the two-dimensional amplitude and phase scan images of a small metal object show a strong response both at the center (feed point) of the antenna, and at the edges of the spectacles’ rims (Fig. 16 left.) However when a dielectric object of the same size is scanned (Fig. 16 right) the response signal only occurs at the center of the antenna where the electric field is maximum. (To aid the eye, the drawing of the antenna has been superimposed on the left hand side images. Photos of the 2 mm objects are added as insets to the top left side of the amplitude images).

In the Critical Dimension (CD) application, the sensor would be called on to image a patterned surface. On the top left of Fig. 17 is shown a test article consisting of a dielectric substrate (polylactic acid or PLA) in which trenches λ/22 wide have been created. Such a structure is similar to the patterned surface that would be inspected at the “Metal 1” integrated circuit manufacturing step^9^ where trenches in SiO_2_ have been etched and are ready to be filled. At the designed optical frequency this would correspond to a 30 nm Critical Dimension case. The raw amplitude image derived by scanning the surface from a distance of 2 mm (equivalent optical distance of 8.3 nm) at a (coarse) 2 mm step is shown on the top right of Fig. 17. On the bottom left is the complement of the pattern where instead of trenches we have dielectric traces or bars. Such structures occur in integrated manufacturing as polysilicon traces on a silicon substrate. The raw amplitude image obtained is shown on the bottom right.

No attempt has been made to sharpen these images further by using signal processing algorithms because that art is well known, and there are many possible strategies that can be adopted depending on the important features of interest.

## Discussion

The capabilities of a massively parallelizable near-field optical sensor for sub-wavelength defect detection and imaging have been demonstrated. Full physics simulations show that the proposed optical sensor, a tuned nano-photonic slot antenna inside a 60 nm slab of silver, is able to detect an object as small as 8 nm. The detection modality is the change in the antenna scattering as a result of the proximity of a sub-wavelength scatterer. Using worst case realistic silver data, we show that this signal can be more than 1.5 orders of magnitude higher than the scattering by the object alone in free space. Therefore this antenna is appropriate for use in the new high signal-to-noise ratio version of the Wave Interrogated Near-Field Array inspection system.

To experimentally validate the simulation results an artificial material was made at radio frequencies to mimic the optical properties of the 60 nm silver slab. Even though this artificial construct violated the conventional rules of effective medium theory, it preserved all the essential physics of the silver slab and its interaction with the slot antenna. The radiofrequency experiments demonstrate the capability of the antenna in detecting λ/78 objects as well as its sensitivity to different materials (dielectric and metal). Using 3D-printed test articles to emulate the “Metal 1” and polysilicon line stages in typical integrated circuit manufacturing, the ability of the sensor to image patterned surfaces with critical dimension of λ/22 was also demonstrated.

## Methods

### Parameters of the FDTD simulation

For the FDTD simulations, an in-house developed code run in parallel FORTRAN was used. The simulation domain was 1200 nm on the side by 400 nm tall and terminated in re-Radiating Boundary Conditions^21^. The illumination source was a Gaussian beam time domain pulse with 200 nm waist. For the sake of simplicity all geometries were assumed to be in air. The presence of a quartz substrate supporting the silver film will of course shift the frequency response but will not change the essence of the results. As explained in the text, the spectacles’ slot antenna design has enough degrees of freedom to tune the frequency to the desired band.

In performing these simulations, the details of the results will vary slightly depending on the model used for the permittivity of the silver layer. For instance Palik’s data^22^ shows more loss than Johnson and Christy’s data^23^. But beyond this, the loss in realistic Silver films also depends on the process for the making of such films. Particularly important are the final surface roughness and the film’s thickness relative to the electron’s mean free path inside the metal. Figure 18 shows the range of silver permittivity data that was used in the simulations. In each case the data over the frequency band of interest was fit with the closest single Drüde term (to match the behavior in the region of interest between 350 nm and 680 nm (blue to red light)) and implemented in the FDTD code using the method of J. L. Young^24^.

It must be mentioned that the material properties are reported using the physics literature convention where the time harmonic factor has been assumed to be e^−iωt^ instead of the engineering convention e^+jωt^. However all other parameters (e.g. circuit parameters) are reported using the engineering conventions in order to elaborate other investigators using the same computational tools (Ansys HFSS) to replicate these results.

### Calculation of the required Admittance sheets to mimic a silver slab

**Figure 6:**
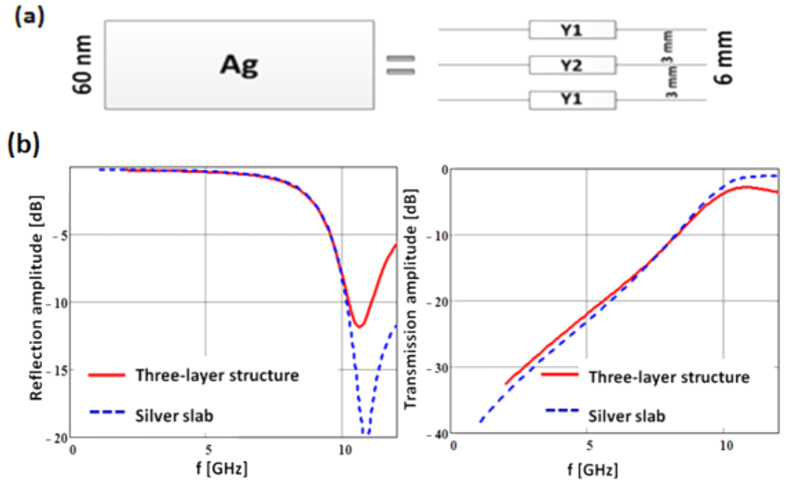
A Silver slab 60 nm thick at 600 nm wavelength can be scaled to a 6 mm slab consisting two dielectric slabs with admittance sheets on their interfaces (**a**). The normal incidence transmission and reflection coefficients are well replicated at and about the frequency of interest.

**Figure 7:**
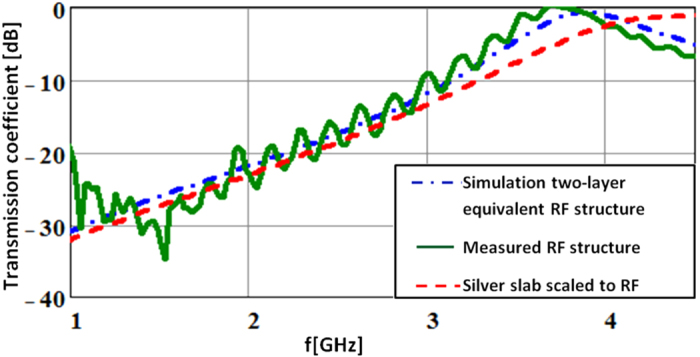
The designed artificial dielectric material structure mimics the transmission properties of silver (with 660 nm scaled down to 2 GHz) from below 1 GHz to approximately 4 GHz.

**Figure 8:**
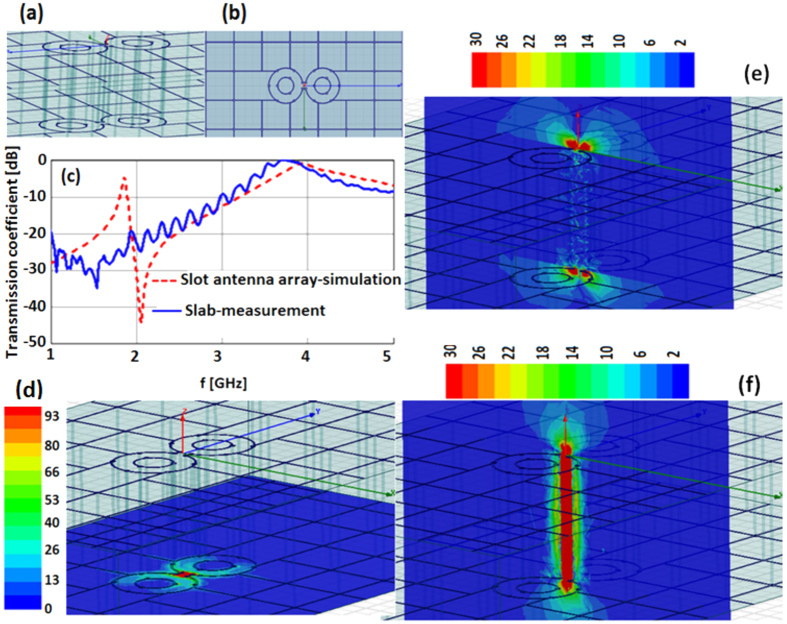
Slot antenna in two-layer grid: perspective view (**a**) and top view (**b**). Transmission through the array of such antenna (simulated) and transmission through the artificial slab (measured) show the extraordinary transmission phenomenon (**c**). Computed results of the field concentration at the open feed of the antenna (**d**) and the transmission of the resonance from the top surface to the bottom surface are seen in the E-field normal to the surface (**e**) and the principal transverse E-field (**f**).

**Figure 9:**
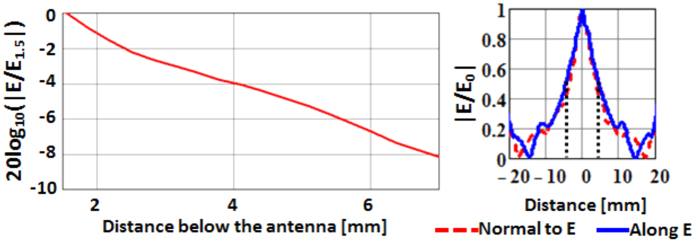
Left: Computed normalized E-field along a line perpendicular to the antenna surface. The factor of normalization, E_1.5_, is the field at 1.5 mm below the antenna. Right: Hot spot of the antenna as normalized magnitude of the total E-field along the two principal planes.

**Figure 10:**
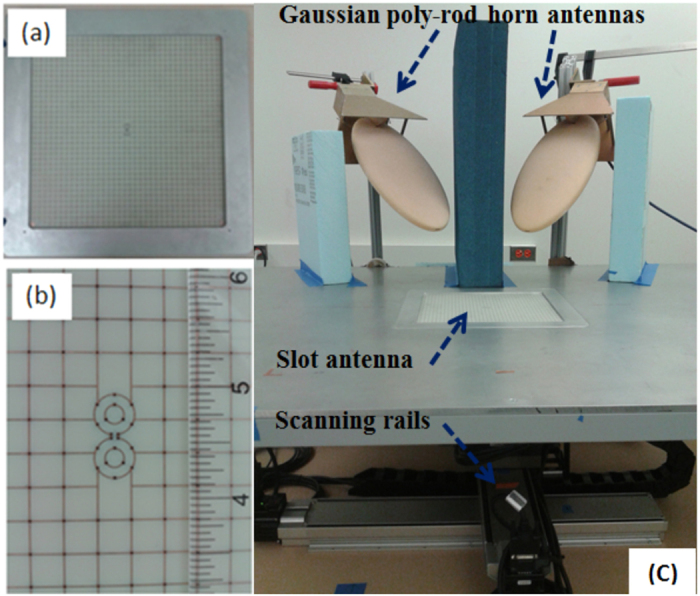
The artificial material slab emulating the 60 nm silver layer at 2 GHz (**a**) contains one antenna (19 mm long by 9 mm wide) at its center. The grid unit cell size is 7.6 mm, the traces are 0.19 mm; the dielectric permittivity is 3.5. (**b**) The plane-wave-interrogated test set up is shown in (**c**). The slot antenna of figure (**a**) is placed at the center of the metal table in (**c**). The Gaussian poly-rod horn antennas are connected to two ports of a vector network analyzer (VNA).

**Figure 11:**
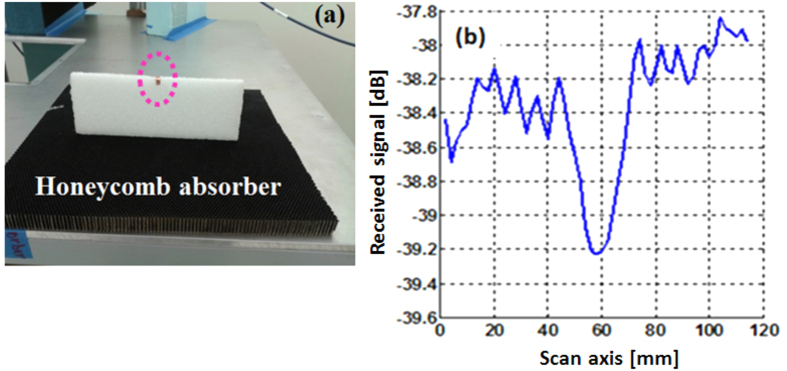
Test object consisting of a metal strip 5 mm wide, held by a piece of Styrofoam on top of a honeycomb absorber (**a**) was scanned along the symmetry plane of the antenna. The received signal for the object approximately 3 mm under the antenna is shown in (**b**).

**Figure 12:**
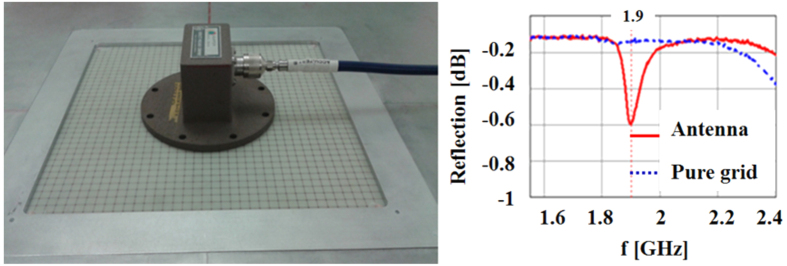
Illuminating the slot antenna with an S band waveguide (left) yields a transmission resonance from the antenna, clearly different from any leakage through the pure grid (right).

**Figure 13:**
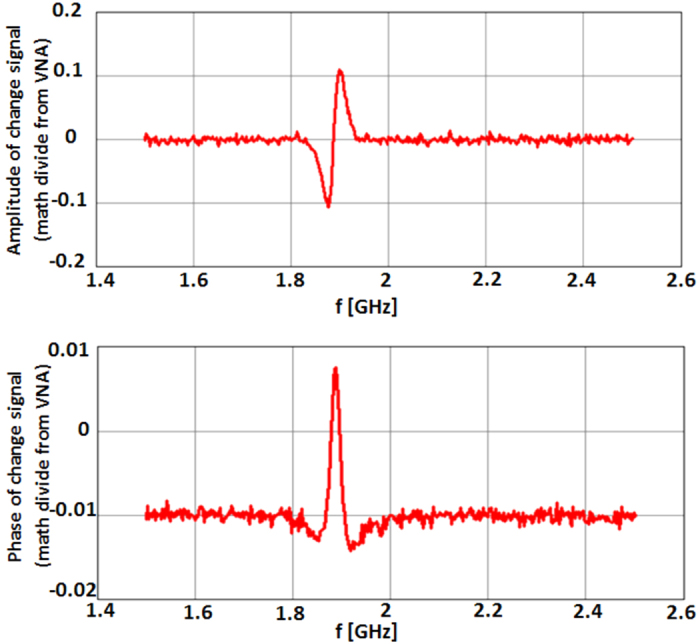
VNA math divide output as the response to a λ/35 object. Top: amplitude of change signal. Bottom: phase of change signal.

**Figure 14:**
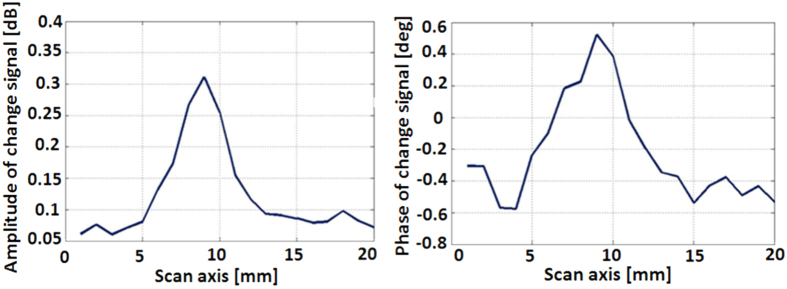
1D scan of a small plastic object (2 mm by 2 mm by 3 mm), that is λ/78 horizontally, with scanning step size of 1 mm. Left: integrated amplitude change signal. Right: integrated phase change signal.

**Figure 15:**
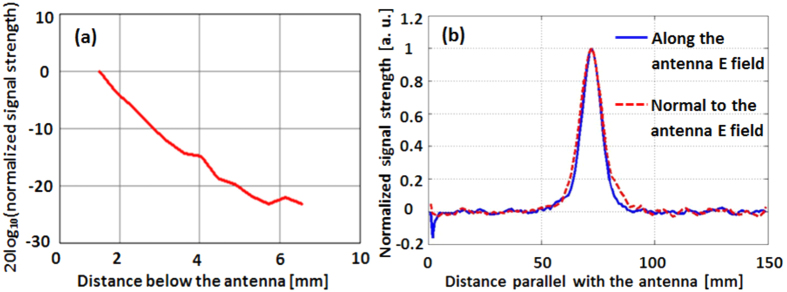
Depth of field (**a**) and hot-spot dimensions (**b**) measured on the RF slot antenna.

**Figure 16:**
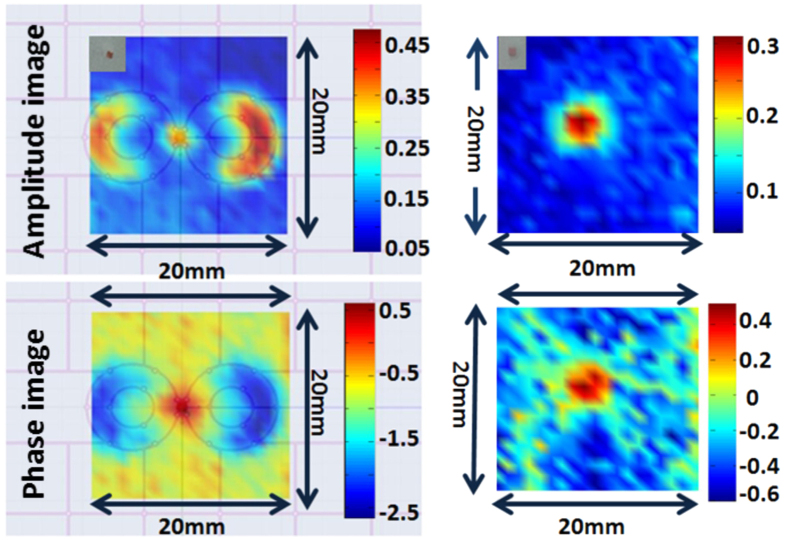
Two dimensional scans of a 20 mm by 20 mm area containing a 2 mm metal (left) and dielectric (right) object.

**Figure 17:**
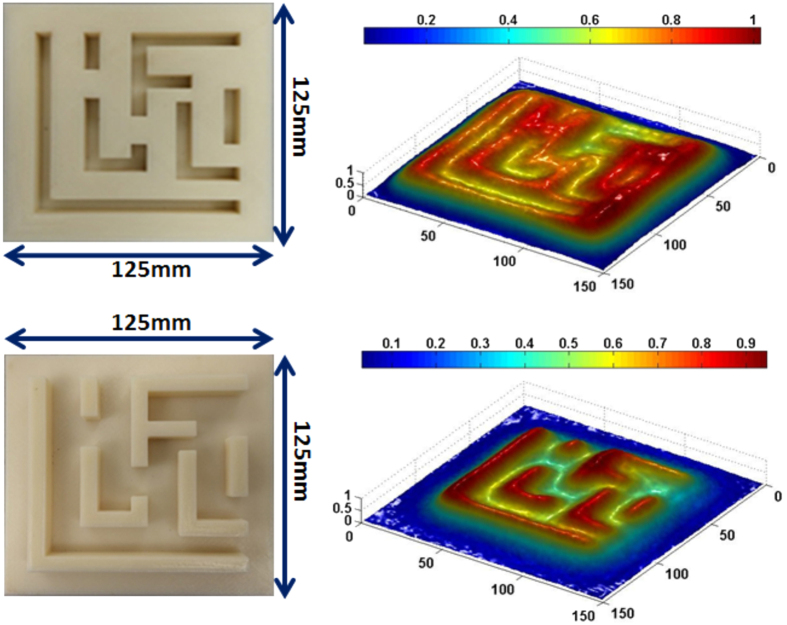
Scans of two different types of patterns emulating the metal 1 trenches step (top) and polysilicon line step (bottom) of integrated circuit manufacturing.

**Figure 18:**
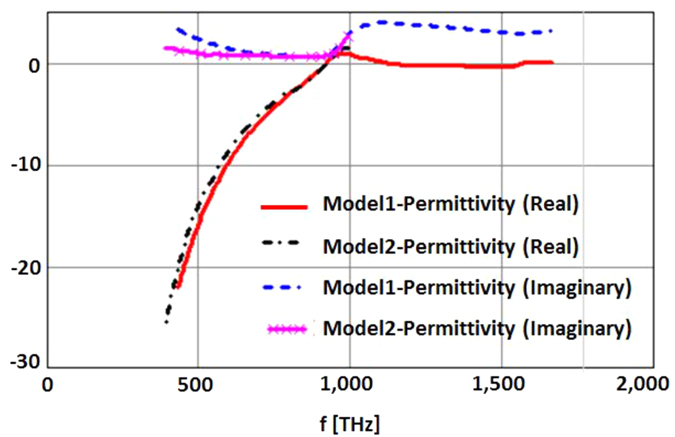
Range of the permittivity models used for the silver data.

Given the desired normal incidence transmission and reflection coefficients, T_*silverslab*_ and Γ_*silverslab*_, and assuming a dielectric substrate of permittivity ε_r_, and thickness, *2t* (in Fig. 6, *t = 3 mm* since that was with the assumption of scaling to 5 GHz), the three admittance surfaces (two Y1, on the outer faces) and Y2 (at the center of the slab) must satisfy equations 4:
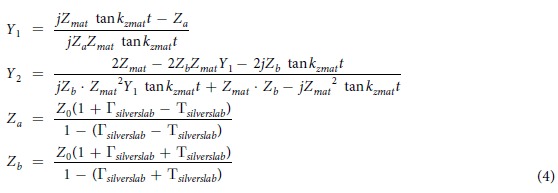
In these equations *Z*_*0*_ is the wave impedance of free space (377 Ω). Defining the propagation constant in free space as *k*_0_ = 2π/*λ*, *k*_*zmat*_ is the propagation constant in the spacing material between admittance surfaces, 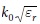 where ε_r_ is the dielectric constant of the material. *Z*_*mat*_ is the wave impedance of the material, 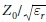. The calculated admittances are then fit to a Lorentz circuit consisting of a series inductance and resistance in parallel with a capacitance, and possibly an additional parallel resistance. In this example, with *ε*_*r*_ = 1, the inductance of Y1 is 6.1 nH, resistance is 25 Ω and capacitance is 30 fF. For Y2, the inductance is 1 nH, the parallel capacitance is 23 pF, and the needed parallel resistance is 700 Ω (that can be ignored in practice).

### Parameters of the HFSS simulations

Frequency domain simulations were performed with Ansys HFSS version 2015.1.0. The structure was placed inside a vacuum box in HFSS. The upper and bottom sides of the box were terminated by Perfectly Matched Layer (PML) boundary conditions and the four lateral sides were assigned periodic boundary conditions. The excitation was a plane wave source.

**Figure 19:**
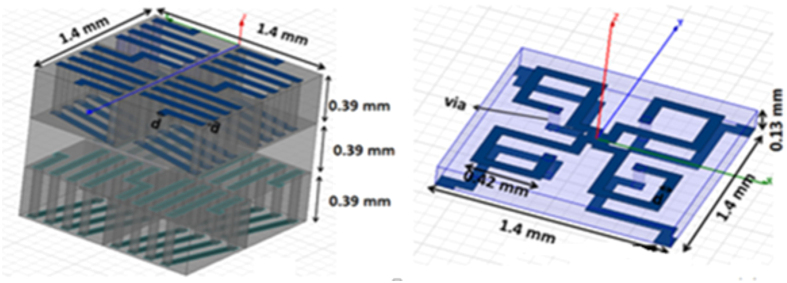
The three layer version of the RF effective medium to simulate the silver slab requires two admittance surfaces. Y1 on the left, Y2 on the right. Dimensions are for scaling to 5 GHz.

Figure 19 shows the HFSS models for the admittance layers of the three layer RF material model, scaled to 5 GHz, using coiled and pancake inductors.

To attain the required inductance these regions cannot be surfaces but have to be expanded into the third dimension as shown. The most important parameter of the admittance circuit to be matched by these constructs is the value of the inductance. To some degree the capacitance can be adjusted independently by fine tuning the width of the traces and by the choice of dielectric constant of the PCB material. However, in the end, the final total slab response is fine-tuned by adjusting the thickness and dielectric constant of the spacer layers separating the admittance surfaces.

The Y1 admittance surface requires a unit cell 1.4 mm on the side each one containing four solenoids (two aligned with the x-axis and two aligned with the y-axis), each solenoid has 9 turns and an approximate cross sectional area of 0.25 mm^2^ implemented using copper traces 0.07 mm wide, spaced 0.07 mm apart, and vias in 0.39 mm thick dielectric layers of ε_r_ = 2.2. The desired inductance is achieved with a parallel capacitance of 84.75 fF.

The Y2 admittance surface located at the center of the slab has the same unit cell and contains 4 pancake inductors, connected diagonally from the center of the unit cell to its four corners. Each pancake has approximately 1.5 turns using both sides of a dielectric layer of ε_r_ = 3.66 and 0.13 mm thick, and also implemented using 0.07 mm wide copper traces and vias. The desired inductance is achieved with a parallel capacitance of 26.8 fF.

### Materials for manufacturing the RF equivalent material and antenna

**Figure 20:**
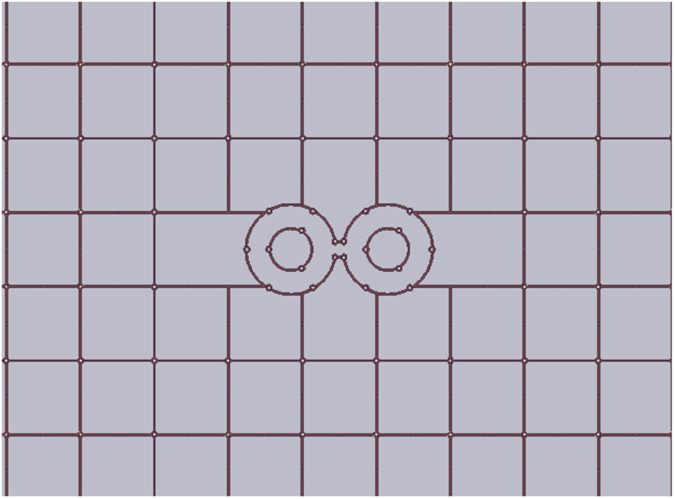
Top and bottom layers of the two-layer grid structure imitating optical transmission and reflection coefficients of a silver slab at 2 GHz, with the slot antenna incorporated into the grid.

The two-layer grid structure was fabricated using Isola FR408 material from Isola Group^©^. The total thickness of the test panel is 14 mm, manufactured using three equal thickness boards. The traces are standard ½ oz. copper. The width of the traces are 0.2 mm, the grid size is 7.6 mm. The via diameter is 0.35 mm and the pad diameter is 0.63 mm. The average radius of the inner circles of the antenna is 2.16 mm and the average radius of the outer circle is 4.6 mm. The middle section of the antenna has the width of 0.905 mm. A close-up view of the top (same as bottom) layer of the two-layer structure with the antenna cut inside is shown in Fig. 20. The surface of the whole grid panel was 304 mm by 304 mm.

## Additional Information

**How to cite this article**: Mostafavi, M. and Diaz, R. E. Feasibility demonstration of a massively parallelizable optical near-field sensor for sub-wavelength defect detection and imaging. *Sci. Rep.* **6**, 26172; doi: 10.1038/srep26172 (2016).
